# Total Extracellular Small RNA Profiles from Plasma, Saliva, and Urine of Healthy Subjects

**DOI:** 10.1038/srep44061

**Published:** 2017-03-17

**Authors:** Ashish Yeri, Amanda Courtright, Rebecca Reiman, Elizabeth Carlson, Taylor Beecroft, Alex Janss, Ashley Siniard, Ryan Richholt, Chris Balak, Joel Rozowsky, Robert Kitchen, Elizabeth Hutchins, Joseph Winarta, Roger McCoy, Matthew Anastasi, Seungchan Kim, Matthew Huentelman, Kendall Van Keuren-Jensen

**Affiliations:** 1 Neurogenomics Division, TGen, 445 N. 5th St., Phoenix, AZ 85004, USA; 2 Department of Molecular Biophysics and Biochemistry, Yale University, New Haven, CT 06520, USA.; 3 Arizona State University Sports Medicine, 323 E Veterans Way, Tempe, AZ 85281, USA; 4 Integrated Cancer Genomics, TGen, 445 N. 5th St., Phoenix, AZ 85004, USA.

## Abstract

Interest in circulating RNAs for monitoring and diagnosing human health has grown significantly. There are few datasets describing baseline expression levels for total cell-free circulating RNA from healthy control subjects. In this study, total extracellular RNA (exRNA) was isolated and sequenced from 183 plasma samples, 204 urine samples and 46 saliva samples from 55 male college athletes ages 18–25 years. Many participants provided more than one sample, allowing us to investigate variability in an individual’s exRNA expression levels over time. Here we provide a systematic analysis of small exRNAs present in each biofluid, as well as an analysis of exogenous RNAs. The small RNA profile of each biofluid is distinct. We find that a large number of RNA fragments in plasma (63%) and urine (54%) have sequences that are assigned to YRNA and tRNA fragments respectively. Surprisingly, while many miRNAs can be detected, there are few miRNAs that are consistently detected in all samples from a single biofluid, and profiles of miRNA are different for each biofluid. Not unexpectedly, saliva samples have high levels of exogenous sequence that can be traced to bacteria. These data significantly contribute to the current number of sequenced exRNA samples from normal healthy individuals.

The field of circulating extracellular molecules is rapidly growing, fueled by the potential for development of new diagnostic and therapeutic tools. As the field is still largely in an exploratory and descriptive phase, there are no standardized methods for sample collection, isolation, or analysis. It is currently unclear what the expectations for a good quality sample should be, and each biofluid under various disease and injury conditions will likely have diverse contents and different criteria for quality. Large datasets examining different biofluids, isolation methods, detection platforms and analysis tools are important to further our understanding of the extent and types of extracellular material present in biofluids. These data will help inform us about how best to develop additional tools to enrich and capture specific types of information.

Circulating extracellular molecular material includes RNAs, DNA, lipids, and proteins (reviewed in refs 1, 2, 3, 4). Carrying these materials to their targets, cells/tissues/organs, and protecting them from degradation, are a variety of extracellular vesicles, lipoproteins, and other RNA-binding proteins^5^,^6^,^7^. A growing number of isolation methods for profiling circulating extracellular molecules have been, and are being, developed. There is still considerable work necessary to identify the most efficient inclusive or selective protocols, depending on the downstream question. There is also a need for rigorous characterization of the biological functions of circulating extracellular RNAs.

There are few large datasets describing the extracellular contents in biofluid samples from normal controls^8^,^9^,^10^,^11^,^12^,^13^. Here we describe the largest dataset to date, focusing on total cell-free RNA (extracellular RNA), using next generation sequencing to profile the small RNA (16–32 nts) payload of human biofluids. Extracellular RNA was isolated and the small RNA content sequenced for 183 plasma samples, 204 urine samples and 46 saliva samples from 55 male college athletes ages 18–25 years. We examined the total RNA in an attempt not to exclude any information in our profile, as extracellular RNAs are packaged not only into extracellular vesicles, but are also associated with lipoproteins^5^ and AGO2^6^,^7^.

We profiled the RNA contents from these samples and examined the prevalence of different RNA biotypes. YRNA and tRNA fragments have previously been identified as abundant RNA species in extracellular vesicles and in biofluids^14^,^15^,^16^,^17^,^18^,^19^,^20^. We also found significant expression of YRNA and tRNA fragments in plasma and urine, respectively. The reasons for such high levels of these small RNA fragments and their functions, are not yet known. YRNA fragments do not appear to be a part of the same regulatory pathway as miRNA or generated by Dicer^21^. They may play a role in apoptosis^18^,^22^, the degradation of misprocessed RNAs^23^, and/or DNA replication^24^. tRNA fragments (TRFs) can be created in response to stress (reviewed in ref. 25). tRNA fragments have also been found to have a unique role in displacing RNA binding proteins that can protect mRNAs from degradation^26^. It should be noted, that tRNAs also have many different modifications along them, which may interfere with their full representation by conventional sequencing approaches^27^,^28^.

miRNAs make up a large portion of the reads generated for each sample. miRNAs typically function in post-transcriptional gene regulation. Their role in extracellular vesicles appears to be less well understood and is potentially more diverse - as a means to rapidly remove miRNA, and as a way to alter local tissue microenvironment and regulate surrounding cells (reviewed in refs 3and 29). We also detect piRNAs that repress transposable elements in the germline (reviewed in ref. 30). It is unknown what role piRNA play in circulating biofluids from normal healthy individuals. We also detect other small RNA species^31^ that are expressed at low levels in biofluids.

Throughout the analysis, it is important to bear in mind that sequencing data provides information on the proportion of RNA biotypes relative to one another in each sample, but not absolute concentrations of RNAs. Therefore, many of the analyses are presented as RPM or percentages of total input reads or reads mapping to the human genome.

## Results

### Summary of the input read alignments

Total cell-free RNA was isolated from plasma, urine, and saliva; sequencing libraries were created using the Illumina TruSeq small RNA preparation kit. We sequenced the small RNA contents from 183 plasma samples, 204 urine samples and 46 saliva samples taken from male college athletes age 18–25. In our comparison of small RNAs in biofluids, we excluded plasma samples with <5% miRNA mapped reads and urine and saliva samples with <0.5% miRNA mapped reads (calculated from the total number of reads mapped to the human genome). The average number of input reads for plasma samples was ~8.9 million reads (median ~8.1 million reads; interquartile range/IQR; 4.7–10.5 million), average ~11.6 million reads for urine samples (median 10.3 million reads; IQR: 7–15.8 million), and an average of ~19 million reads for saliva samples (median 14.8 million reads; IQR: 10.9–22.6 million). Accordingly, 161 plasma, 159 urine, and 30 saliva samples fit these criteria and were included in the forthcoming analyses.

**Figure 1:**
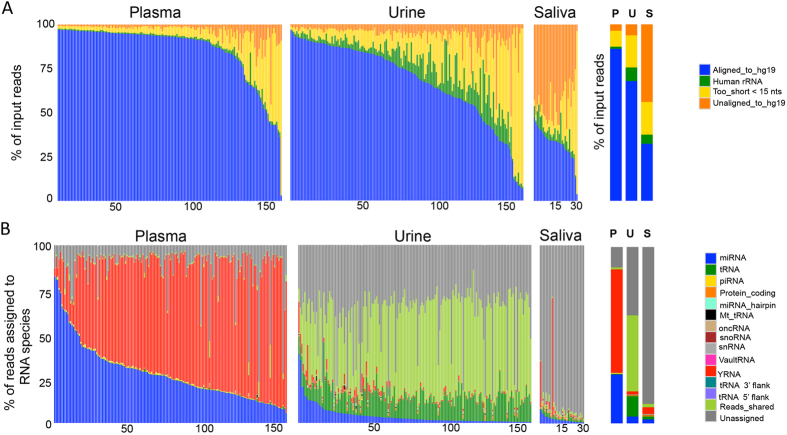
Distribution of total input reads and reads mapped to the genome. (**A**) Displays the alignment of the input reads for each biofluid to the human genome, human rRNA, reads that were too short (<15 nts), and unaligned to the human genome. (**B**) Displays the distribution of the reads mapped to the human genome to RNA biotypes: miRNA, tRNA, piRNA, protein-coding fragments, miRNA hairpins, Mt_tRNA (mitochondrial tRNA), oncRNA (other non coding RNA), snoRNA, snRNA, Vault RNA, YRNA, tRNA flanking regions, 3′ and 5′ (50 bps flanking the mature tRNA sequence), more than one RNA biotype, and reads that are unassigned (intergenic, intronic, and overlapping with >40 regions to the genome).

Figure 1A displays the percentage of input reads that aligned to the human genome (blue), aligned to human rRNA (green), were too short after adaptor removal (<15 nucleotides; yellow), or did not align to the human genome (orange) for each sample in the analysis. A detailed sample list with the percentage of reads in each category can be found in Supplementary Table S1. After removal of reads that were too short and reads that mapped to human rRNA, the average number of reads aligned to the human genome for plasma samples were 86% (median 93%: IQR 85–96%), 68% for urine (median 74%; IQR: 56–86%), and 32% for saliva (median 34%; IQR: 28–36%). The percentage of rRNA is highest in the urine samples with an average of 8% (median 6%), followed by saliva samples with an average of 5% of the reads (median ~4%), and plasma with 1% of the total input reads mapping to rRNA (median <1%). The average percentage of reads that are too short (<15 nucleotides) in the saliva samples are ~18% (median 8%), in urine samples it is ~18% (median 11%), and in plasma samples it is 9% (median 4%). In the saliva samples, a median of ~45% of the reads are unaligned to the human genome.

### Summary of small RNA biotypes

We next examined the percentage of reads assigned to abundant RNA categories or RNA biotypes (Fig. 1B). The reads that remain after mapping to rRNA and miRNA are mapped simultaneously to mature tRNAs, mature piRNAs and to all other RNA transcripts in GENCODE (Ensembl 75). This alignment strategy leads to a large percentage of read sequences that are identical in more than one RNA biotype. These sequences that overlap exactly with more than one RNA biotype, form the most abundant category of reads in the urine samples. The light green bars in Fig. 1B, depicting ‘reads shared’ across more than one biotype or position in the genome, constitute ~54% of the reads from urine samples and ~3% of aligned reads in saliva samples. In urine, these reads are primarily shared between tRNA and piRNA. Supplementary Table S2 provides a complete list of each sample and the percentage of reads going to each RNA biotype.

The RNA biotype that is most represented in plasma samples is YRNA, with a median of 63% of the reads mapped (IQR 52–73%). A more detailed analysis of YRNA fragments is discussed in a section below. Plasma samples have the highest percentage of reads assigned to miRNAs, when compared to urine and saliva, with a median of 25% (IQR: 17% to 34%; Wilcoxon p value <1.5E-12). The urine samples have 2% of their reads assigned to miRNA (IQR of 1–4%) and saliva 1% reads are assigned (IQR 2–3%). A more complete statistical comparison of small RNA biotypes across and within biofluids, can be found in Supplementary Table S3. The grey bar denoted as “unassigned” refers to reads that do not have a known gene annotation, such as intergenic or intronic regions of the genome. This category also includes reads that multi-map to more than 40 different annotations. Saliva samples have the highest percentage of reads (~93%) that are unassigned.

### Multiple alignments of short reads

There are several challenges to small RNA sequence analysis that influence the final categorization of the reads into biotypes. Many short read sequences are shared by more than one RNA biotype, making it difficult/impossible to resolve to a single unique RNA classification for many of the reads. The substantial percentage of reads shared amongst different RNA biotypes, when mapped concurrently, motivated us to examine these analyses more thoroughly. As was described previously, we allowed the reads to align across biotypes simultaneously; after removing reads that aligned to rRNA and miRNA. Similar analysis recommendations are outlined by the NIH Extracellular RNA Communication Consortium (ERCC) in Freedman *et al*.^10^.

**Figure 2:**
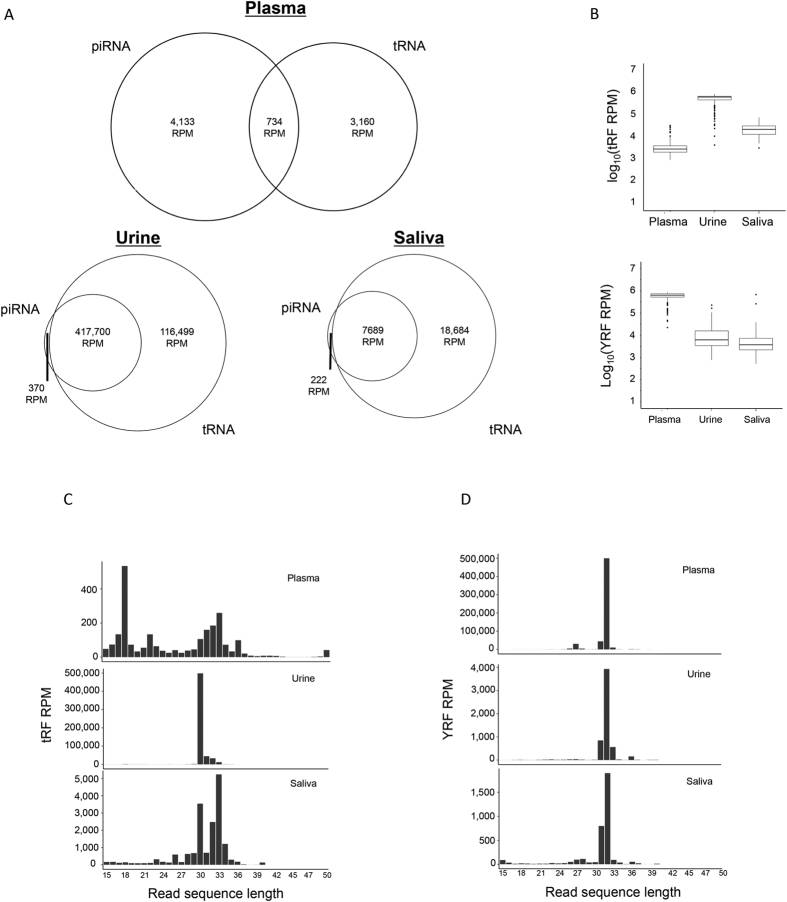
Reads aligning to tRNA and piRNAs. (**A**) Read overlap between piRNA and tRNA. A large number of sequences detected by sequencing simultaneously align to both piRNA and tRNA. We assessed the distribution of reads per million mapped to the human genome and the numbers that were uniquely classified as piRNA, uniquely classified as tRNA, or overlapped between the two RNAs. In urine and saliva samples, there were few reads that exclusively mapped to piRNA. This does not rule out the presence of piRNA, but the origin of these sequences would have to be further investigated. (**B**) The upper and lower panels display the number of tRNA and YRNA fragments displays the number of tRNA fragments normalized as reads per million mapped to the human genome (RPM) found in each biofluid. Urine has very high levels of tRNA fragments compared to plasma and saliva, normalized as reads per million mapped to the human genome (RPM) found in each biofluid. Urine has very high levels of tRNA fragments compared to plasma and saliva and the lower panel demonstrates that there are a large number of YRNA fragments found in plasma compared to urine and saliva. (**C** and **D**) These two panels display the lengths for the tRNA and YRNA fragments respectively, identified in each biofluid and their abundance.

We observed a substantial percentage of read sequences shared between tRNAs and piRNAs, particularly in the urine samples. Figure 2A displays Venn diagrams for plasma, urine and saliva samples that depict the average RPM (reads per million are calculated from reads mapped to the human genome), for reads mapping only to tRNAs, only to piRNAs and the reads mapping to both these RNA biotypes simultaneously for all three biofluids. Evident from the Venn diagrams, plasma samples have the lowest proportion of read sequences shared between tRNAs and piRNAs, 734 RPM on average shared between these two RNA biotypes. The saliva samples have on average 7,689 RPM mapping simultaneously to both tRNAs and piRNAs and 18,684 RPM mapping to tRNAs only. The urine samples have on average 417,700 RPM shared between tRNAs and piRNAs and an additional ~116,500 RPM aligning solely to tRNA fragments. Only 370 RPM align to piRNAs, therefore, the small RNA profile of the urine samples appears to be dominated by tRNA fragments.

Based on the sequence of the piRNAs in piRBase and the mature tRNA sequences, we find that there are 133 piRNAs that share sequences with less than or equal to 2 mismatches. For example in the urine samples, the top 5 piRNA sequences that share the largest number of reads with tRNAs are piR-hsa-1207, piR-hsa-28131, piR-hsa-24672, piR-hsa-5937, piR-hsa-5938. The sequences for the first two piRNAs differ by only one base (piR-hsa-1207: AGCATTGGTGGTTCAGTGGTAGAATTCTCGC, piR-hsa-28131: GGCATTGGTGGTTCAGTGGTAGAATTCTCGC), and overlap with the 5′ end of 10 Gly tRNAs at an average RPM of 411,839 GCATTGGTGGTTCAGTGGTAGAATTCTCGC.

The last 3 piRNAs differ by only 1–2 bases (piR-hsa-24672: TTCCCTGGTGGTCTAGTGGTTAGGATTCGGC, piR-hsa-5937: TCCCTGGTGGTCTAGTGGTTAGGATTCGGCA, piR-hsa-5938: TCCCTGGTGGTCTAGTGGTTAGGATTCGGCAC), these overlap with the 5′ end of 8 Glu tRNAs sequences detected at RPM of 4730. There were 26, 22, and 25 piRNA sequences, that did not overlap with other RNA biotypes, consistently detected in 80% of the plasma, urine, and saliva samples respectively. These piRNA sequences shared no overlap with tRNAs.

### Detailed tRNA fragments analysis

The presence of tRNA fragments cleaved from mature tRNAs in cells as a response to stress is known^32^,^33^,^34^,^35^,^36^,^37^,^38^. Recently tRNA fragments (tRF) have also been shown to be present in extracellular RNA in biofluids^15^,^17^,^19^. Mature tRNAs contain a number of post transcriptional modifications which hinder efficient sequencing by NGS^27^,^28^. A complete list of modifications can be found at the Modomics website (http://modomics.genesilico.pl/sequences/list/). Thus, the detection of tRNA fragments in sequenced samples can arise due to 2 main reasons: a) biologically induced tRNA cleavage, b) inability of the reverse transcriptase to process the entire tRNA strand due to the presence of modifications. Because we did not sequence these samples specifically with the removal of RNA modifications, we cannot distinguish between a) and b). Regardless, the tRNA fragments that were detected using our sequencing protocols in urine samples exceed those found in saliva and plasma (Wilcoxon p-value < 1.5E-15; Fig. 2B upper panel). The median tRF RPM for plasma, urine and saliva are 2,912, 624,387 and 22,103 respectively (Wilcoxon p-value < 2.2E-16). Figure 2C represents the length distribution of the tRF reads. In the plasma samples, there is a bimodal distribution of the reads with two clear peaks at 18 nts and 30–33 nts, the latter of which is found in the saliva samples. In the urine samples, there is a sizeable well-defined single peak at 30 nts. The presence of this peak at ~500,000 RPM is two and three orders of magnitude higher than the saliva and plasma samples respectively for their highest expressed tRF. The location of these tRFs was examined on the mature tRNA, whether it originates from the 5′ (tRF5), 3′ (tRF3) or neither end (tRFM, tRNA fragment middle). Table 1 summarizes the median percentage of reads originating from tRF5, tRF3 and tRFM. Figure S1A shows the percentages for individual tRNA fragments across all samples for all three biofluids as stacked bar plots. The percentages are calculated based on the total number of reads assigned to tRNAs, after the samples have been normalized for library size using median ratio normalization (DESeq2; ref. 39).

Table 2 summarizes the top ten expressed tRFs by amino-acid type for the three biofluids with the median percentage assigned shown in brackets. The rest of the tRNAs are combined and referred to as “Other tRNAs”. tRNAs belonging to the Gly-GCC family are the highest expressed in all three biofluids, followed by Glu-CTC in urine and Val-CAC in plasma and saliva. These are the sequences detected using conventional sequencing, and the presence of modifications may be masking other abundant tRNAs. Supplemental Figure S1B displays the percentage of reads assigned to different tRNAs based on the amino-acid type and the top 10 tRNA.

### Detailed YRNA fragments analysis

According to ENSEMBL 75, there are 4 human YRNAs; RNY1, RNY3, RNY4, RNY5. There are an additional 52 transcripts which are pseudogenes based on the 4 human YRNAs and a further 878 predicted YRNA transcripts make up the YRNA category. As mentioned before, in the plasma samples ~63% of the reads assigned to the human genome align to YRNAs in our samples. The presence of YRNA fragments has been reported previously^16^,^17^,^18^,^19^. Figure 2B (lower panel) illustrates the proportion of reads aligning to YRFs in plasma exceeds those found in saliva and urine (Wilcoxin p-value < 1.3E-16). The median YRF RPM, based on reads mapped to the human genome, for plasma, urine and saliva are 629,023, 6,224 and 3,735 respectively. Figure 2D displays the length distribution of the YRF reads. Unlike the tRFs, the YRFs have a unimodal distribution with a single large peak at 32 nts in all three biofluids. The majority of the YRNA fragments originate from the 5′ end. Table 3 summarizes the median percentage of reads arising from the YRF5, YRF3 or YRFM (middle) for all three biofluids. The percentages are calculated based on the total number of reads assigned to YRNAs, after the samples have been normalized for library size by the median ratio method of normalization. The most abundant YRF originates from the 5′ end of RNY4. It is responsible for a median 93%, 97% and 84% of the RPM assigned to YRFs in the plasma, urine and saliva samples respectively. The length of the most abundant YRF is 32, which maps to 9 YRNA annotations in ENSEMBL 75- RNY4, 2 YRNA pseudogenes, and 6 genes predicted by RFam.

### Detailed miRNA analysis

**Figure 3:**
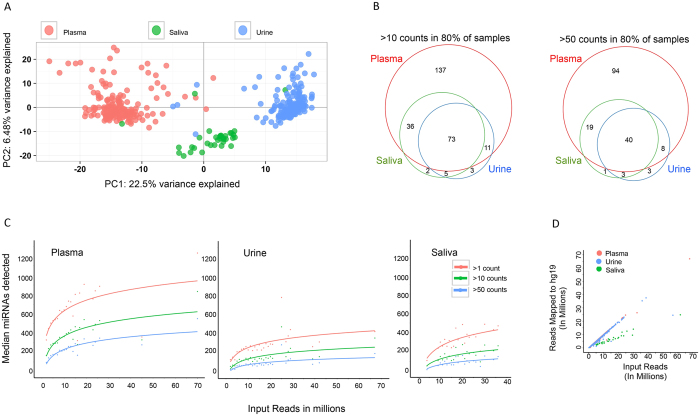
Distribution of miRNAs in biofluids. Panel A is a principal components analysis of the miRNAs detected in each biofluid. Each biofluid has a distinct miRNA pattern. Panel B displays the miRNAs detected in each biofluid with >10 or >50 counts in at least 80% of the samples. There are only a handful of miRNAs uniquely detected in urine and saliva at this level of expression. Most miRNAs can be detected in plasma. (**C**) shows the number of detected miRNAs at 1 count, 10 counts or 50 counts, as a function of input reads. (**D**) shows the number of reads mapped to the human genome as a function of the input reads. Saliva samples require larger numbers of input reads to achieve the same numbers of reads aligned to the genome as plasma and urine. Urine samples behave similarly to plasma samples with respect to input reads that map to the genome (**D**), but have fewer miRNAs detected (**C**).

As most laboratories focus their analysis on the miRNA contents of biofluids, we examined characteristics of this small RNA biotype in greater detail. A principal component analysis (PCA) of the miRNAs from each biofluid demonstrates that samples cluster primarily by biofluid type, Fig. 3A. The top ten miRNAs with the highest absolute loadings for the first principal component (PC1) were hsa-miR-30a-5p, hsa-miR-1273h-3p, hsa-miR-30a-3p, hsa-miR-30c-2–3p, hsa-miR-10b-5p, hsa-miR-199a-5p, hsa-miR-204-5p, hsa-miR-4433b-5p, hsa-miR-6852-5p and hsa-miR-126-3p. The top ten miRNAs with the highest absolute loadings for the second principal component (PC2) were hsa-miR-320a, hsa-miR-26b-5p, hsa-miR-421, hsa-miR-29a-3p, hsa-miR-450b-5p, hsa-miR-155-5p, hsa-miR-26a-5p, hsa-miR-30c-5p, hsa-miR-32-5p and hsa-miR-361-5p. The first and second principal components cumulatively explain ~29% of the variance in all the samples.

We examined the most robust miRNAs for each biofluid, requiring detection with >10 or >50 read counts in 80% of the samples for each biofluid. Most miRNAs are detectable in plasma samples, a few unique miRNAs are detectable in saliva and urine (Fig. 3B). Summarized in Table 4 are the number of miRNAs detected with at least 10 or 50 counts and the number of samples in which they are found for each biofluid. From Table 4, there are 975 miRNAs detected in at least one of the sequenced plasma samples with >10 counts. If we examine miRNAs that are consistently detected in plasma samples, we find 329 miRNAs expressed >10 counts in 50% of the samples and only 98 miRNA detected in 100% of the plasma samples. 545 miRNAs are detected in at least one urine sample, and 122 miRNAs are identified at >10 counts in 50% of samples, and 25 miRNAs with >10 counts were found in 100% of the samples. 336 miRNAs were detected at least once in a saliva sample with >10 counts, and 141 and 69 miRNAs were detected with >10 counts in 50% and 100% of saliva samples. There are surprisingly few miRNAs consistently detected in all samples. Supplementary Table 4 summarizes the miRNAs detected in all samples. In this table, we describe the number of samples in which each miRNA was detected, for each biofluid. We also display the level of expression for that miRNA in each biofluid.

#### Limit of detection of miRNAs

The analysis for detection of miRNAs in Table 4 depends on read depth and the complexity of miRNAs and other small RNAs in the sample. Each library loaded onto the sequencer has some variability in read depth, and the amount of rRNA and other RNA biotypes present in the sample can alter the number of reads that align to the genome and to miRNA. Using some of the libraries loaded with higher than expected read depth, we can calculate how many miRNAs are detected with increasing library size. Figure 3C displays the number of miRNAs observed as a function of input reads. The samples are binned by one million read increments. The median number of detected miRNAs for each bin with greater than 1, 10 and 50 read counts are plotted. As expected, the solid line depicts the increase in the number of miRNAs detected as a logarithmic function with respect to the sequencing depth. For the plasma samples, an increase in the sequencing depth from 10–11 million reads to greater than 20 million reads adds another 64 miRNAs that are detected with at least 50 read counts. However, for the urine samples, a commensurate increase in sequencing depth adds only 17 miRNAs detected with 50 counts. For saliva, doubling the sequencing depth from 4–6 million reads to greater than 10 million reads adds 78 miRNAs detected with at least 50 counts. Saliva samples require many more input reads to achieve meaningful levels of reads mapped to the human genome, and therefore to the detection of miRNAs (Fig. 3D).

#### miRNAs with highest and lowest CVs

We next wanted to assess how similar the samples within each biofluid were to each other, and if the samples were taken from the same individual, were they more similar than when compared to the samples from the whole group. We examined data from individuals that provided more than 5 samples over time (11 individuals provided >5 plasma samples and 5 individuals provided >5 urine samples). It should be noted, the samples obtained from an individual were not equally distributed in time, a collection period could span 69 weeks. We did not have >5 saliva samples sequenced from any individuals, and therefore did not include an analysis. The miRNA read counts for all samples were normalized for library size using the median ratio method.

**Figure 4:**
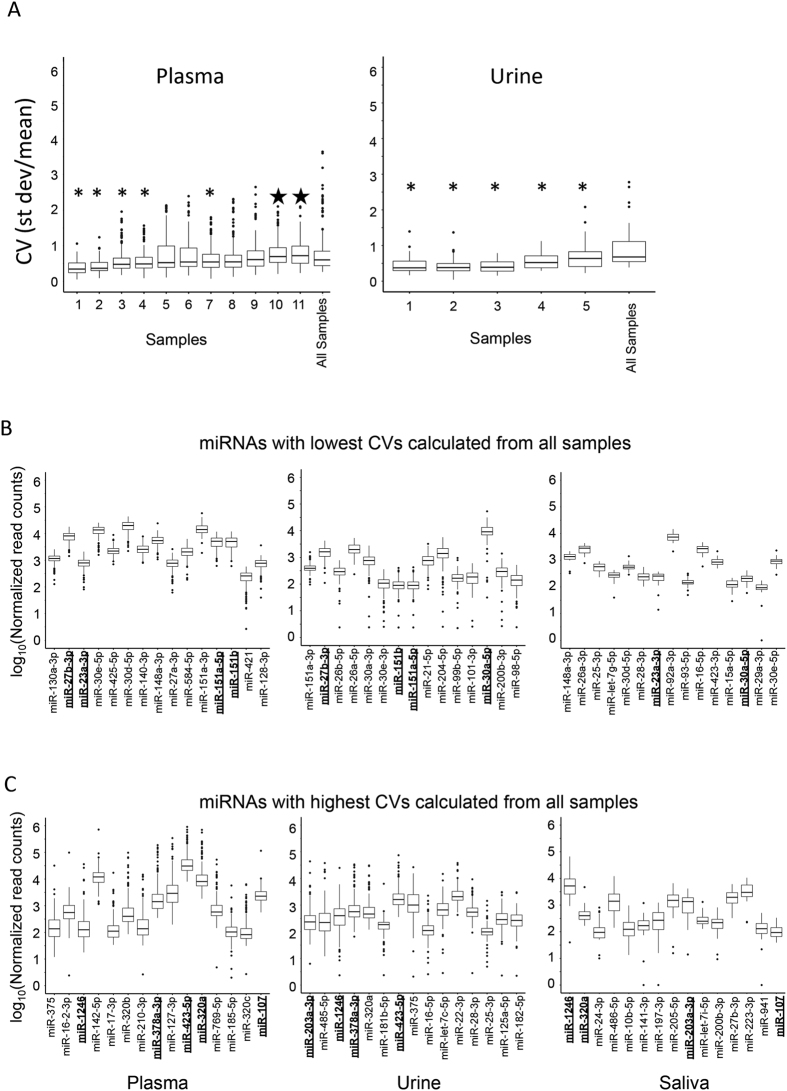
Coefficient of variation for multiple samples taken from the same individual compared with the coefficient of variation from all subjects. (**A**) displays the distribution of CVs calculated for each miRNA detected at >50 counts in at least 80% of the plasma and urine samples. Some of the individual subjects^1^,^2^,^3^,^4^,^5^,^6^,^7^,^8^,^9^,^10^,^11^ that provided more than 5 samples for sequencing over the course of ~70 weeks, show a closer distribution of CVs when compared to samples from all subjects (asterisk). And some of the subjects with >5 samples display a higher CV than when examining all subjects at once (star). Distribution of miRNA counts for the 15 miRNAs with the lowest CV (**B**) and the highest CV (**C**) in each biofluid. At this time, we are unable to determine if the CVs are related to biological variability or to technical variability.

The coefficient of variation (CV) was calculated to assess the dispersion of miRNA expression among all of the samples sequenced, and multiple samples collected from the same individuals. In this analysis we included only well-expressed miRNAs, >50 read counts and detected in at least 80% of the samples. The box plots in Fig. 4A display the distribution of miRNA CV values for each individual sampled at least 5 times. The distribution of the miRNA CVs for the “All samples” boxplot takes into account one sample per individual. The boxplots for the individuals depict intra-individual variance and the last boxplot for “All samples” represents inter-individual variance. For urine, the intra-individual miRNA variation is significantly less than the inter-individual variation. A two-sided Wilcoxon rank sum test reveals that this difference is statistically significant, with p-values < 0.05 for all individuals and p-value < 0.001 for individuals 1, 2, 3 and 4 (See Fig. 4A). miRNAs detected in the plasma samples had a wider range of variability, higher (star) and lower (asterisk) CV than the samples from all individuals.

Figure 4B,C display the 15 miRNAs with the lowest CVs (4B) and highest CVs (4C) for all three biofluids across all samples sequenced. miRNAs that were in common between at least two biofluids are bolded and underlined, only one miRNA was highlighted for all biofluids. miR-1246 had a high CV in all biofluids. Box plots in Supplementary Figure S2 display the top 15 miRNAs with the lowest CV and highest CV for individuals (5 miRNAs overlap in the analysis of the lowest CVs and 5 miRNAs overlap in the calculated highest CVs).

### Detailed exogenous RNA analysis

We did not directly target exogenous RNA sequences in our samples. However, we assessed the potential bacterial content in the samples using GOTTCHA; Genomic Origin Through Taxonomic CHAllenge^40^. GOTTCHA is a profiling tool that uses read-based metagenome characterization following a hierarchical collection of exclusive signatures at multiple taxonomic levels such as strain-level, species, genus, family, order, class and phylum. Owing to similarity in genomic regions, such as 16S rRNA, coding regions and other highly conserved regions, metagenomic identification typically yields results with a high false discovery rate (FDR). We used the precompiled bacterial database available at ftp://ftp.lanl.gov/public/genome/gottcha/ consisting of unique species-level genomic signatures that was produced by eliminating shared 24-mer (k24) sequences from 4937 bacterial replicons (includes both chromosomes and plasmids) and the human genome, while retaining a minimum of 24 bp of unique fragments. When mapped to the species-level database, the reads are rolled up to the next higher taxonomic orders, which are genus, family, order, class and phylum. Further elimination of false positives entail that the organism discovered must have a minimum of 100 non-overlapping bases covering the unique genomic signature, a coverage of at least 0.5% and at least 10 hits to the unique signature. These stringent requirements allow for the identification of bacteria in the biofluid samples with a potentially low false positive rate. Out of the 161 plasma exRNA samples sequenced, only 9 samples had at least one significant species of bacteria detected. Only two species of bacteria was seen in 4 or more samples: *Candidatus Tremblaya princeps* and *Brucella melitensis*. One sample had 315 species of bacteria present, with the highest read counts going to *Desulfovibrio vulgaris, Pyrococcus furiosus, Singulisphaera acidiphila, Rhodopirellula baltica, Asticcacaulis excentricus* and *Synechococcus sp.* WH 8102 *Desulfobacca acetoxidans*.

66 out of the 159 urine exRNA samples had at least one significant species of bacteria detected. Escherichia coli was seen in 7 samples and only three species of bacteria was seen in >25% of the 66 samples: *Achromobacter xylosoxidans, Gardnerella vaginalis* and *Streptococcus pneumonia*, with 22, 20 and 17 samples respectively. One sample had 1433 species of bacteria present; with the highest read counts going to *Prevotella intermedia, Singulisphaera acidiphila, Micrococcus luteus, Arthrobacter phenanthrenivorans* and *Amycolicicoccus subflavus*. A large number of bacterial species are seen in the saliva samples.

There are 110 bacterial species seen in at least 22 of the 30 saliva samples (75% of the saliva samples sequenced). The top ten most highly detected bacteria are: *Rothia mucilaginosa, Prevotella melaninogenica, Dyadobacter fermentans, Streptococcus salivarius, Methanolacinia petrolearia, Bacillus megaterium, Anaerobaculum mobile, Singulisphaera acidiphila* and *Rothia dentocariosa*. In the saliva samples, an average of ~45.5% of the reads mapped uniquely to bacterial species (after adapter trimming and removing reads that were <15 nts). Table 5 provides a snapshot of the species most commonly detected and in what number of samples. Supplementary Table S3 has a more thorough analysis of the bacterial species detected using GOTTCHA.

## Discussion

This is the largest dataset examining extracellular RNA expression, and the presence of different RNAs, to date. As we are deciphering what quality metrics differentiate a good sample from a bad sample – and what should be expected from extracellular RNA samples, more data sets are required. Different isolation, sequencing, and analysis tools will need to be applied to large data sets so that we can accurately gauge the true signature from technical artifact. In addition, more data exploring a significant range of age and gender will be important. The Extracellular RNA Communication Consortium (ERCC) is actively developing an extensive atlas for normal extracellular RNAs from biofluids, and from several diseases and injuries. The dataset described here will be deposited in the atlas where it can be compared with other samples examining extracellular RNAs (the data can also be found in dbGaP, accession # phs001258.v1.p1).

We analyzed this data for several key abundant RNA biotypes, however, there are several more biotypes that could be explored using this data^9^,^10^. Each biofluid appears to have clear differences in extracellular RNA expression profiles. For example, there appears to be a high proportion of tRNA fragments in urine samples, when compared with other RNA biotypes. In particular, there is one fragment that has very high read counts. It would be interesting to know if the tissues in closest proximity to urine (kidney, bladder, adrenal gland) had very high levels of this tRNA or the fragment. Or, perhaps the increased proportion of the fragments in urine is due to some filtering mechanism for that biofluid. Better small RNA tissue atlases that include more comprehensive profiles of the small RNA species will be necessary to help answer these questions.

Unique sequences that align exclusively to piRNA in urine and saliva samples are very low. It is not possible to say that there are no piRNA in these biofluids, but their origin and overlap with other RNA biotypes will have to be carefully examined.

We assessed YRNAs, which accounted for ~63% of the reads mapping to the genome for plasma samples. It is unclear what the role these 5 prime YRNA fragments have in normal healthy individuals, but they are found in significant abundance. The Gingeras laboratory recently observed that RNY5 fragments, found in vesicles isolated from cancer cells, could trigger cell death^18^. Urine (0.7%) and saliva (0.5%) samples did not show such high levels of YRNA fragments.

miRNAs are the most diverse RNA biotype found in biofluid samples. Most of the miRNAs could be detected in plasma, with few unique miRNAs in urine and saliva. While almost 1000 miRNAs could be detected in all plasma samples, if we required higher stringency - that they be detected in most samples (80%) with at least modest expression levels (>10 counts), the number of miRNAs in the analysis went down dramatically. Because sequencing does pick up such large numbers of other small RNAs (YRNA, tRNA, etc.), a more targeted approach to miRNA detection may reveal a larger number of miRNAs consistently expressed.

Not surprisingly, saliva had a large number of reads going to bacterial species. The largest category of reads aligned to the human genome for the saliva samples was, unassigned, meaning that most reads were to intergenic, intronic or were sequences that mapped to >40 places in the genome and could not be assigned to any location. More reads actually aligned to bacterial species than to the human genome. We used an algorithm, GOTTCHA, to detect bacteria in our samples^40^. Urine samples had some bacterial species identified, and only a handful of samples had a large diversity of bacteria detected. This analysis was interesting and showed few plasma samples that had detectable levels of bacteria. There was one plasma sample that had 315 bacterial species detected at low levels, likely due to contamination. In future experiments, additional negative controls should be examined to verify that the bacterial species came from the biofluid samples, and not contamination from subject skin, collection process, isolation or kit preparations.

As we examine extracellular RNA profiles in biofluids, we are looking for consistent RNAs that can be detected with confidence in each biofluid, as well as normal levels of expression for comparison to disease and injury. We found that miRNAs from urine, that were assessed more than 5 times from the same individuals over a years time frame, were closer together than when examining the miRNA dispersion in samples taken from all subjects. This was not the case for plasma samples, where some individuals had higher and lower variability over time than when compared to all subjects. This may indicate that establishing a baseline for individuals when they are healthy may provide the most meaningful comparisons when exploring early indicators of disease, severity, or outcome. While we cannot determine at this time if the lowest and highest CVs are due to technical or biological variability, we believe this is worth keeping track of as more large datasets become available. For example, miR-1246 was found to have one of the highest CVs in each biofluid. Does this miRNA reflect rapid turnover and changes in response to biological events, or significant technical variability due to sample collection, handling or sequencing? As more large datasets examining the full profile of extracellular RNAs found in biofluids emerge, it will be important to learn what variables alter the detection of RNAs. As more individuals share their samples, analysis and classification schemes, other researchers can improve upon the methods to increase accuracy and remove variability from the analysis. Therefore, we have tried to provide the most comprehensive profile of the most abundant RNA species detected in our samples using current tools and databases. This information will be essential as the field moves toward using these expression changes for the detection of health, disease, and injury.

## Materials and Methods

### Samples

Samples were collected from male college athletes ages 18–25. All human subjects provided written consent form prior to enrollment. All samples were collected with consent and approval from the Western Instiutional Review Board (WIRB) study ID# 1307009395. Small RNA profiling experiments from human samples were performed at the Translational Genomics Research Institute (TGen) in accordance with the regulations and proper approval from WIRB. Blood samples were collected in EDTA tubes and placed in a cooler with ice packs until they were transported from Arizona State University (ASU) to TGen, within 2–3 hours of blood draw. Samples were spun down at 2500 RPM for 10 minutes at 4 °C. Plasma was aliquotted at 1 mL volumes into 2 mL RNase/DNase free Microcentrifuge tubes (VWR), and stored at −80 °C. Urine was collected in sterile cups and placed in a cooler with ice, and transported to TGen within 2–3 hours of collection. Samples were spun at 3000 RPM for 10 minutes at 4 °C and aliquotted 15 mL into a 50 mL conical tube for storage at −80 °C. Saliva samples were collected by allowing passive drool to collect and spitting into a 50 mL conical tube. The sample was spun at 3000 RPM for 10 minutes at 4 °C, aliquotted as 1 mL volumes into 2 mL microcentrifuge tubes and stored at −80 °C.

### RNA Isolation

For all plasma and saliva samples we isolated 1 mL of biofluid. Samples were isolated using the *mir*Vana miRNA Isolation Kit (ThermoFisher Scientific, AM1560) according to Burgos *et al*., 2013^41^. Samples were DNase treated using TURBO DNA-free Kit (ThermoFisher Scientific, AM1907). Because of residual phenol/chloroform, samples were then cleaned and concentrated using Zymo RNA Clean and Concentrator (Zymo Research, R1016) using Protocol: Purification of small and large RNAs into separate fractions and combining the fractions at the end. All urine samples (15 mL) were isolated using Urine Total RNA Purification Maxi Kit, Slurry Format (Norgen Biotek Corp., Cat#29600). Samples were DNase treated on column using RNase-Free DNase Set (Qiagen, cat# 79254). Because there was no residual phenol/chloroform, samples were concentrated by Speed Vacuum.

### Sequencing

All sequencing data is available through the ERCC exRNA Atlas and through accession number phs001258.v1.p1 in dbGaP. The plasma, saliva and urine RNA were quantified in triplicate using Quant-iT Ribogreen RNA Assay kit, Low-Range protocol (R11490; ThermoFisher). The Illumina small RNA TruSeq kit (RS-200–0048; Illumina) was used for sequencing all samples. RNA input for plasma and saliva was 10–20 ng for all samples and the RNA input for urine was 30 ng for all samples. The reagents from the Illumina TruSeq kit were halved, as in Burgos *et al*., 2013. Each sample was assigned one of 48 possible indices. We used 16 PCR cycles for all samples. Indexed samples were run on a gel and purified away from the adaptor band. The samples were then pooled and placed on Illumina V3 single read flowcells (GD-401-3001; Illumina). The average read counts at each nucleotide length for each biofluid is displayed in Supplementary Figure S3.

### RNA-Seq data analysis

The raw sequence image files from the Illumina HiSeq 2500 in the form of bcl are converted to the fastq format using *bcltofastq* v1.8.4 and checked for quality to ensure the quality scores do not deteriorate drastically at the read ends. The adapters from the 3′ end are clipped using *cutadapt* v.1.10 (http://cutadapt.readthedocs.io/en/stable/guide.html). Reads shorter than 15 nts are discarded and after adapter trimming, the 3′ bases below a quality score of 30 are trimmed as well. All subsequent steps are carried out using *sRNABench* (http://bioinfo5.ugr.es/sRNAbench/sRNAbench.php), which provides an elegant framework to map the reads to various RNA libraries using Bowtie1 to perform the alignments. The reads are first mapped to human rRNA sequences obtained from NCBI and those that map are removed from analysis. The algorithm used in *sRNABench* is based on the *mirAnalyzer*^42^, where the reads with the same sequence are collapsed and mapped to the human genome and miRNA database. A single base mismatch and a seedlength of 19 nts are used for this step. The reads that remain after mapping to the miRNA database are then mapped to mature tRNAs, piRNAs and all other RNAs in ENSEMBL 75. Here, there is no mismatch allowed and each read is allowed to multimap to at most 40 RNA annotations. Table 6 provides the list of libraries used and their versions.

### Analysis of the tRNA fragments (tRFs)

All reads that map to mature tRNAs are used for the analysis. The reads, based on their read sequence are stacked as shown in Table 7 and the read counts for all reads that share the same 5 prime or 3 prime end are added up for that particular tRNA. For example, the following fragments arise from the 5′ end of mature tRNA GluCTC. There are 8 mature GluCTC tRNAs with identical 5′ ends. These fragments are now “collapsed” to their longest sequence and all read counts are added up (bottom row).

The fragments that arise from the 5 prime and the 3 prime end of the mature tRNAs are termed tRF5s and tRF3s respectively. Fragments that arise from neither of these two ends are termed tRFMs (tRF middle). This gives rise to a list of fragments with unique sequences whose source on the mature tRNA (tRF5, tRF3 and tRFU), the length distribution and the type of tRNA fragment (ValCAC, GlyGCC, etc) are now known.

### Analysis of YRNA fragments

Analysis was carried out in the same manner as tRNA analysis.

### Analysis of the exogenous bacterial species

The precompiled bacterial database available at ftp://ftp.lanl.gov/public/genome/gottcha/ consisting of unique species-level genomic signatures that was produced by eliminating shared 24-mer (k24) sequences from 4937 bacterial replicons (includes both chromosomes and plasmids) and the human genome, while retaining a minimum of 24 bp of unique fragments was used. All the parameters used for filtering out false positives were the default parameters in GOTTCHA.

## Additional Information

**How to cite this article:** Yeri, A. *et al*. Total Extracellular Small RNA Profiles from Plasma, Saliva, and Urine of Healthy Subjects. *Sci. Rep.* **7**, 44061; doi: 10.1038/srep44061 (2017).

**Publisher's note:** Springer Nature remains neutral with regard to jurisdictional claims in published maps and institutional affiliations.

## Supplementary Material

Supplementary Tables

Supplementary Figures

## Figures and Tables

**Table 1:** tRNA fragment analysis.

Biofluid	tRF5	tRF3	tRFM	Reads Assigned to tRNAs
**Plasma**	38%	30%	28%	20,643
**Urine**	99%	0%	1%	5,403,970
**Saliva**	89%	1%	10%	75,387

The number of fragments arising from the 5′, 3′ or middle of the tRNA sequence. The percentages are calculated based on the number of reads assigned to tRNAs after normalizing each sample for library size by the median ratio method. The median percentage of all samples is reported here for tRF5, tRF3, tRFM. The last column is the median number of reads assigned to tRNAs for each biofluid.

**Table 2:** Percentage of reads assigned to the top 10 detected tRNA in each biofluid.

Plasma	Urine	Saliva
GlyGCC (18%)	GlyGCC (86%)	GlyGCC (24%)
ValCAC (10%)	GluCTC (5%)	ValCAC (24%)
ValAAC (9%)	ValCAC (3%)	GluCTC (11%)
ProCGG (9%)	GluTTC (0.6%)	GluTTC (3%)
GluCTC (8%)	GlyCCC (0.5%)	ValAAC (3%)
GlnTTG (5%)	MetCAT (0.4%)	LysCTT (3%)
ProTGG (3%)	LysTTT (0.4%)	LeuCAG (2%)
GlnCTG (3%)	ProCGG (0.2%)	HisGTG (2%)
LysCTT (2%)	HisGTG (0.2%)	LysTTT (1%)
GluTTC (2%)	ValAAC (0.1%)	AlaAGC (1%)
Other tRNAs (18%)	Other tRNAs (0.6%)	Other tRNAs (8%)

The tRNA detected in each biofluid are listed and the percentage of the reads for each are shown in parentheses. It is important to note that what is described here are the results using conventional sequencing techniques, RNA modifications may prevent full profiling for some of the top tRNA.

**Table 3:** YRNA fragments originating from the 5′, 3′, or middle section of the YRNA sequence.

Biofluid	YRF5	YRF3	YRFM	Reads assigned to YRNAs
**Plasma**	93%	0%	7%	5,156,001
**Urine**	98%	0%	2%	41,114
**Saliva**	84%	0%	16%	9,377

The percentages are calculated based on the number of reads assigned to YRNAs after normalizing each sample for library size by the median ratio method. The median percentage of all samples is reported here for YRF5, YRF3, YRFM. The last column is the median number of reads assigned to YRNAs for each biofluid.

**Table 4:** Number of miRNAs detected in each biofluid.

	Plasma Detected miRNA		Urine Detected miRNA		Saliva Detected miRNA	
	>10 counts	>50 counts	>10 counts	>50 counts	>10 counts	>50 counts
Detected in at least 1 sample	975	585	545	299	336	144
Detected in 10%	472	271	191	102	200	107
Detected in 20%	396	237	171	87	179	95
Detected in 30%	363	211	146	80	163	86
Detected in 40%	340	201	131	70	154	84
Detected in 50%	329	190	122	66	141	79
Detected in 60%	306	177	110	64	132	76
Detected in 70%	287	171	104	59	123	72
Detected in 80%	257	162	92	54	116	63
Detected in 90%	213	132	79	46	103	59
Detected in 100%	98	82	25	19	69	37

The number of miRNAs detected >10 or >50 counts in at least one sample of plasma, urine, or saliva samples, or in 10, 20, 30, 40 − >100% of samples.

**Table 5:** List of exogenous species detected by GOTTCHA.

Biofluid	TAXA	Number of samples	Average RPM
Plasma	Brucella melitensis	5	2.6
	Candidatus Tremblaya princeps	4	17.5
	Thermus sp. CCB_US3_UF1	3	45.2
	Candidatus Tremblaya phenacola	3	29.2
	Propionibacterium acnes	2	313.9
	Synechococcus sp. PCC 6312	2	21.0
	Pseudomonas monteilii	2	38.2
	Streptococcus suis	2	22.5
	Sphingomonas sp. MM-1	2	6.1
	Listeria monocytogenes	2	6.5
Urine	Achromobacter xylosoxidans	22	1655.1
	Gardnerella vaginalis	20	376.1
	Streptococcus pneumoniae	17	147.1
	Streptococcus suis	16	56.6
	Thermus sp. CCB_US3_UF1	16	22.6
	Candidatus Tremblaya phenacola	14	26.4
	Salmonella enterica	12	36.3
	Candidatus Tremblaya princeps	12	23.7
	Microcoleus sp. PCC 7113	10	282.7
	Cyanothece sp. PCC 7425	10	179.8
Saliva	Rothia mucilaginosa	29	3623.6
	Streptococcus salivarius	29	1894.9
	Prevotella melaninogenica	29	2400.2
	Dyadobacter fermentans	15	4544.9
	Bacillus megaterium	19	1440.5
	Methylacidiphilum infernorum	19	1513.4
	Methanolacinia petrolearia	17	2164.8
	Vibrio vulnificus	19	911.6
	Sulfobacillus acidophilus	14	1386.6
	Singulisphaera acidiphila	14	1658.6

List of exogenous species detected by GOTTCHA. The 10 most prevalent bacterial species (detected in the largest number of samples) for each biofluid, are displayed. The number of samples the bacteria was detected in and the expression (RPM = reads mapping to the unique bacterial genomic signature divided by the input number of reads > = 15 nts) are shown.

**Table 6:** Database of RNA biotypes used.

Library type	Version and notes
rRNA	Mt_rRNA sequences from Ensembl 75 (ENST00000389680, ENST00000387347), rRNA sequences for the 12S rRNA (gi|407595|gb|S64650.1), RNA5-8S5 (NR_003285.2), RNA5S4 to RNA5S17, RNA18S5 (NR_003286.2), RNA28S5 (NR_003287.2), RNA45S5 (NR_046235.1)
miRNA	miRBase v.21. Consists of 2587 human miRNA entries
tRNAs	gtRNAdb, hg19. Consists of 624 entries. The mature tRNA sequences were obtained after removing the introns using tRNAscan-SE (Lowe, T.M. and Eddy, S.R. (1997) Nucleic Acids Res, 25: 955–964.). After removal of introns, the bases “CCA” were added to the 3′ end as would be expected for a mature tRNA (tRFdb: A database for transfer RNA fragments, Nov, 2104). Also, 50 bp flanking both the 3′ and the 5′ were included
piRNAs	pirBase v.1.0. Consists of 32827 human piRNAs
All RNA from Gencode	hg19, ENSEMBL 75Consists of the following RNA biotypes:Protein coding, Mt-tRNA, Mt-rRNA, snoRNA, snRNA, VaultRNA, YRNA and other non-coding RNA. This latter category is a collective for 3prime_overlapping_ncRNA, antisense, IG_C_gene, IG_C_psuedogene, IG_D_gene, IG_J_gene, IG_J_psuedogene, IG_V_gene, IG_V_psuedogene, lincRNA, LRG_gene, misc_RNA, polymorphic_psuedogene, processed_psuedogene, psuedogene, sense_intronic, sense_overlapping, TR_C_gene, TR_D_gene, TR_J_gene, TR_J_psuedogene, TR_V_gene, TR_V_psuedogene
Sequence	Read Counts

**Table 7:** Example for the collapse of tRNA sequences.

Sequence	Read Counts
TCCCTGGTGGTCTAGTGGTTAGGATTCGGCGCTCTCACCG	95
TCCCTGGTGGTCTAGTGGTTAGGATTCGGCGCTCTCACC	3
TCCCTGGTGGTCTAGTGGTTAGGATTCGGCGCTCTCAC	5
TCCCTGGTGGTCTAGTGGTTAGGATTCGGCGCTCTCA	6
TCCCTGGTGGTCTAGTGGTTAGGATTCGGCGCTCTC	3,752
TCCCTGGTGGTCTAGTGGTTAGGATTCGGCGCTCT	14,528
TCCCTGGTGGTCTAGTGGTTAGGATTCGGCGCTC	13,967
TCCCTGGTGGTCTAGTGGTTAGGATTCGGCGCT	68,915
TCCCTGGTGGTCTAGTGGTTAGGATTCGGCGC	329,486
TCCCTGGTGGTCTAGTGGTTAGGATTCGGCG	50,571
Collapsed Sequence	Total
TCCCTGGTGGTCTAGTGGTTAGGATTCGGCGCTCTCACCG	482,479
