# Supplementary material for: Total Extracellular Small RNA Profiles from Plasma, Saliva, and Urine of Healthy Subjects
Source: Sci Rep. 2017 Mar 17;7:44061. doi: 10.1038/srep44061 (PMC5356006; doi:10.1038/srep44061)
Supplement: Supplementary Figures [file srep44061-s2.pdf]

Total Extracellular Small RNA Profiles from Plasma, Saliva, and Urine of Healthy Subjects
Ashish Yeri[1], Amanda Courtright[1], Rebecca Reiman[1], Elizabeth Carlson[1], Taylor Beecroft[1], Alex Janss[1], Ashley Siniard[1], Ryan Richholt[1], Chris Balak[1], Roger McCoy[2], Matthew Anastasi[2], Seungchan Kim[3], Matthew Huentelman[1] and Kendall Van Keuren-Jensen[1]*
[1]Neurogenomics Division, TGen, 445 N. 5th St., Phoenix, AZ 85004
[2]Arizona State University Sports Medicine, 323 E Veterans Way, Tempe, AZ 85281
[3]Integrated Cancer Genomics, TGen, 445 N. 5th St., Phoenix, AZ 85004
* Corresponding author

Supplemental Figure S1

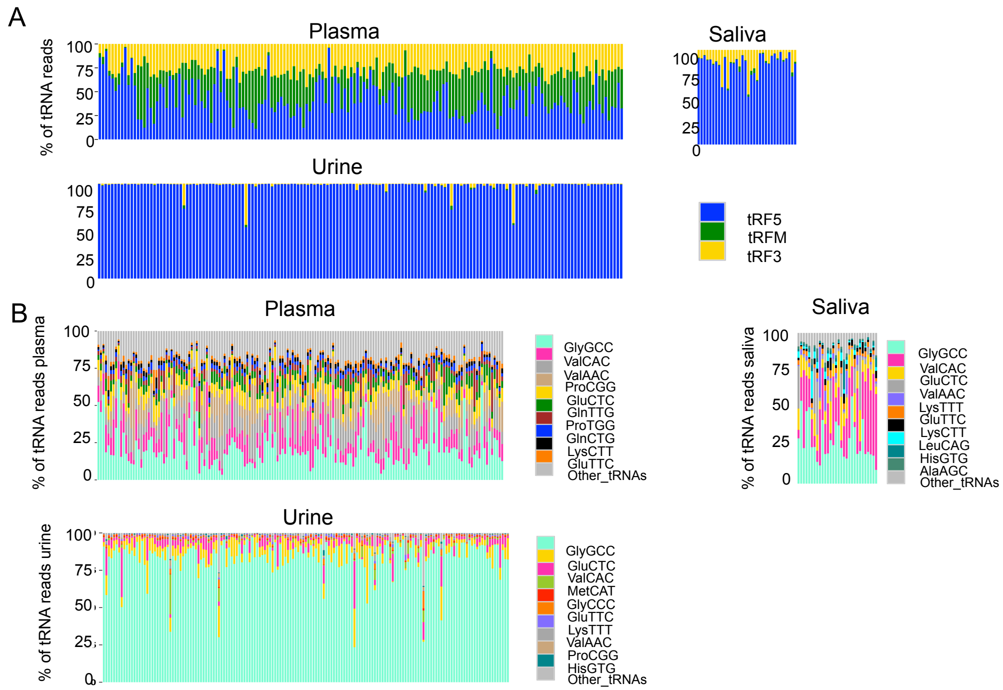


**Supplementary Figure S1. Detailed analysis of the detectable tRNA fragments.** Of the reads that map to tRNA fragments, (A) shows the number that aligns to the 5', 3', or middle of tRNA (tRF5, tRF3, or tRFM) for each biofluid. The fragments aligning to different detected tRNAs are shown in B.

Supplemental Figure S2

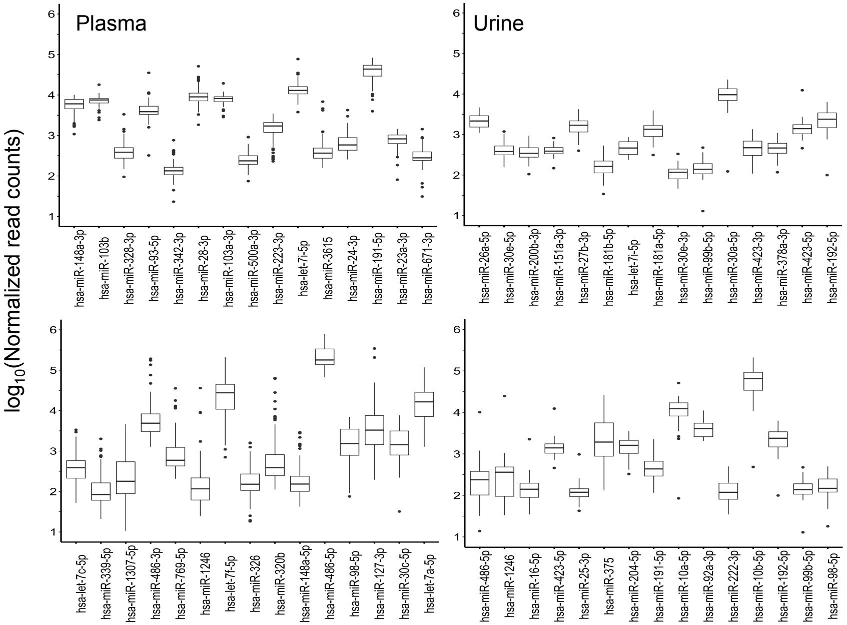


**Supplementary Figure S2. CVs for miRNAs from individuals with more than 5 samples sequenced over ~70 weeks.** (A) displays the 15 miRNAs with the lowest CVs, and (B) displays the 15 miRNAs with the highest CVs).

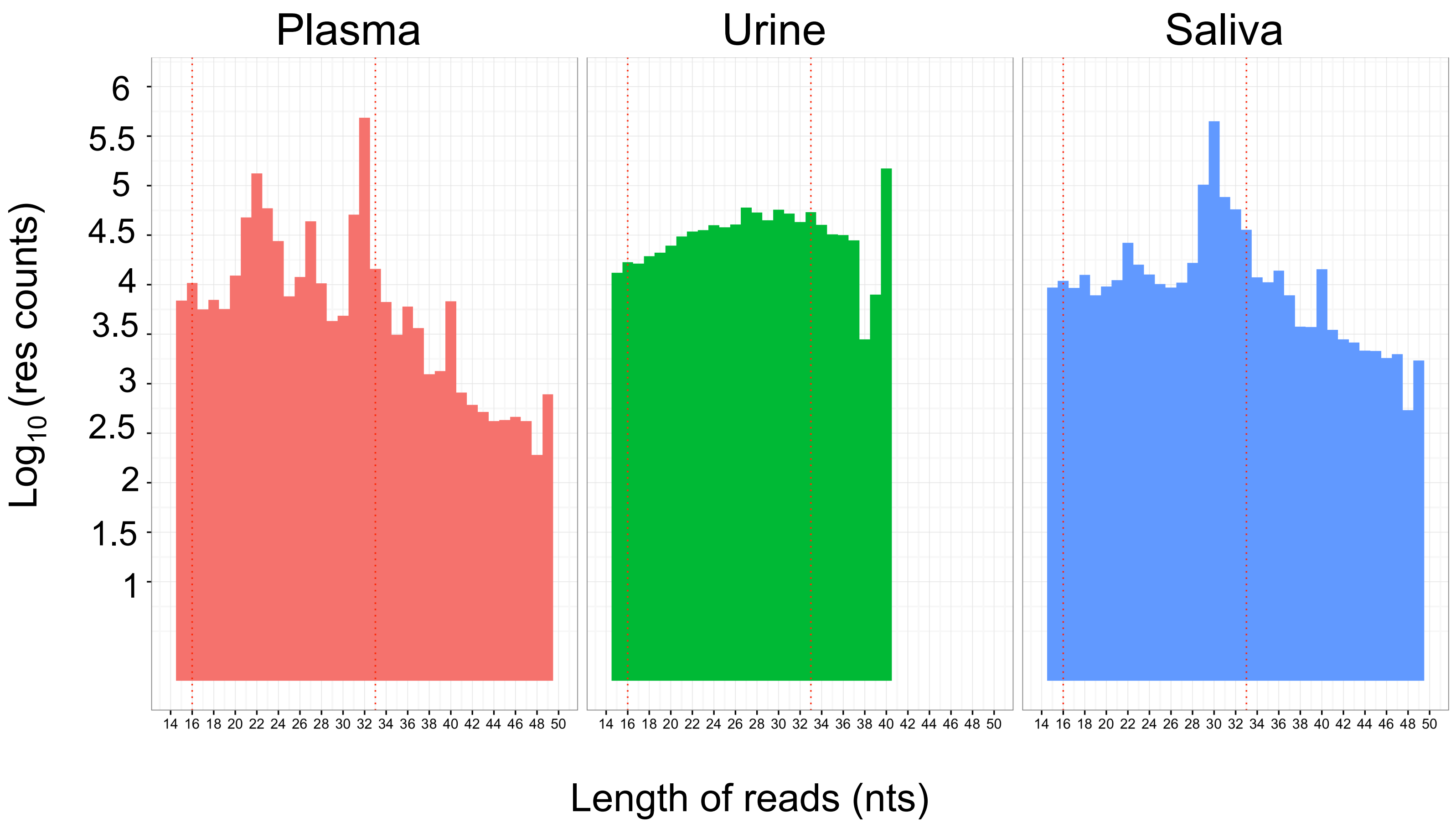


**Supplementary Figure S3. Read length distribution for plasma, urine and saliva samples. The dotted red line represents the range of RNAs discussed in the manuscript. miRNAs typically have a length of 16-23 nts and the tRNA and yRNA fragments discussed in the manuscript are between 30-33 nts long.**
